# Combined Longitudinal Effect of Physical Activity and Screen Time on Food and Beverage Consumption in European Preschool Children: The ToyBox-Study

**DOI:** 10.3390/nu11051048

**Published:** 2019-05-10

**Authors:** María L. Miguel-Berges, Alba M. Santaliestra-Pasias, Theodora Mouratidou, Pilar De Miguel-Etayo, Odysseas Androutsos, Marieke De Craemer, Sonya Galcheva, Berthold Koletzko, Zbigniew Kulaga, Yannis Manios, Luis A. Moreno

**Affiliations:** 1 Growth, Exercise, Nutrition and Development (GENUD) Research Group, University of Zaragoza, C/Pedro Cerbuna 12, 50009 Zaragoza, Spain; albasant@unizar.es (A.M.S.-P.); theodoramouratidou@icloud.com (T.M.); pilardm@unizar.es (P.D.M.-E.); lmoreno@unizar.es (L.A.M.); 2 Instituto Agroalimentario de Aragón (IA2), 50009 Zaragoza, Spain; 3 School of Health Science (EUCS), University of Zaragoza, C/Domingo Miral s/n, 50009 Saragossa, Spain; 4 Centro de Investigación Biomédica en Red de Fisiopatología de la Obesidad y Nutrición (CIBERObn), 50009 Zaragoza, Spain; 5 Department of Nutrition and Dietetics, School of Health Science and Education, Harokopio University, 17671 Athens, Greece; oandrou@hua.gr (O.A.); manios.toybox@hua.gr (Y.M.); 6 Department of Movement and Sports Sciences, Ghent University, Watersportlaan 2, 9000 Ghent, Belgium; marieke.decraemer@ugent.be; 7 Department of paediatrics. Medical University Varna, 55 Marin Drinov Str., 9002 Varna, Bulgaria; sonya_galcheva@mail.bg; 8 Dr von Hauner Children’s Hospital, University of Munich Medical Centre, 80337 Munich, Germany; berthold.koletzko@med.uni-muenchen.de; 9 The Children’s Memorial Health Institute, 04-730 Warsaw, Poland; z.kulaga@ipczd.pl

**Keywords:** screen time, physical activity, preschool children, food and beverage consumption

## Abstract

Lifestyle behavioral habits such as excess screen time (ST), a lack of physical activity (PA), and high energy-dense food consumption are associated with an increased risk of children being overweight or obese. This study aimed to (1) track longitudinal adherence to PA and ST recommendations at baseline (T0) and follow-up (T1) and (2) assess the association between changes in adherence to PA and ST recommendations and food and beverage consumption at follow-up. The present study included 2321 preschool children (3.5 to 6) participating in the multicenter ToyBox-study. A lineal mixed effects model was used to examine the association between different types of food and beverages and their relationship with changes in adherence to PA and ST recommendations. Approximately half of the children (50.4%) did not meet the PA and ST recommendations at both baseline and follow-up. However, only 0.6% of the sample met both PA and ST recommendations. Preschool children who met both recommendations consumed fewer fizzy drinks, juices, sweets, desserts, and salty snacks and consumed more water, fruits and vegetables, and dairy products than did those not meeting both recommendations. In conclusion, the proportion of European preschool children adhering to both PA and ST recommendations was very low and was associated with a low consumption of energy-dense foods.

## 1. Introduction

Being overweight or obese during childhood and adolescence is a major public health challenge [1]. Excess weight gain during childhood is associated with long-term health risks and adult diseases such as cardiovascular disease, type 2 diabetes, and hypertension [2]. Behaviors such as excessive screen time (ST), a lack of physical activity (PA), and high consumption of energy-dense foods have been shown to be independently associated with increased risks of being overweight or obese in children, adolescents, and adults [3]. Individually and combined, high sugar-sweetened beverage and low fruit and vegetable consumption are associated with an increased obesity risk [4,5], while behaviors such as PA appear to be protective [6,7]. Hence, it is crucial that interventions during childhood target lifestyle behaviors such as diet, physical activity, and sedentary behavior, which are established in early years and track into adulthood [8].

PA guidelines for preschool children recommend that preschool children should spend at least 180 min per day doing PA [9]. Limited evidence suggests that a total daily physical activity volume of 10,000–14,000 steps per day is associated with 60–100 min of moderate vigorous PA in preschool children [10]. De Craemer et al. [11] proposed using 11,500 steps per day as an attainable and realistic cut-off for PA recommendations, helping to promote PA among preschool children. Regarding ST, established guidelines for preschool children (one- to five-year-olds) state that they should limit TV viewing and use of other electronic media such as computers, DVDs, and other electronic games to less than one hour per day [9].

ST has been shown to be associated with increased energy-dense food and beverage consumption and decreased fruit and vegetable (F & V) consumption in preschool children [12]. In European children participating in the IDEFICS study, low time spent on moderate to vigorous physical activity (MVPA) was associated with a low consumption of vegetables and yogurt and high fast food consumption [13]. A low socioeconomic status was also associated with consumption of high energy-dense foods, increased ST, and low levels of PA [14,15].

To the authors’ knowledge, there are no studies that have investigated the individual and combined effects of PA and ST on food consumption in preschool children. For this reason, the current study aimed to (1) track longitudinal adherence to PA and ST recommendations at baseline (T0) and follow-up (T1) and (2) assess the association between changes in the adherence to PA and ST recommendations and food and beverage consumption at follow-up (T1).

## 2. Methods

### 2.1. Study Design

The ToyBox-study (www.toybox-study.eu) was were a cluster-randomized clinical trial aiming to prevent obesity in preschool children. It was conducted in six European countries, namely Belgium, Bulgaria, Germany, Greece, Poland, and Spain. The detailed protocol is described elsewhere [16,17]. In total, 309 kindergarteners and 7056 children aged 3.5–6 years were recruited at baseline (T0), and 5529 children continued at follow-up (T1) [18]. The ToyBox intervention aimed to promote preschool children’s water consumption, healthy snacking, and PA and limit/interrupt their sedentary time by improving the children’s physical and social environment both in kindergarten and at home. In this study, 2321 (33% of the baseline sample) preschool children were included with complete information from a parental questionnaire and also pedometer information at baseline (T0) and follow-up (T1). Data collection was carried out in May–June 2012 (T0) and May–June 2013 (T1). The ToyBox-study adhered to the Declaration of Helsinki and the conventions of the Council of Europe on human rights and biomedicine. In all countries, ethical approval was obtained from their respective ethical committees and local authorities.

### 2.2. Socioeconomic Variables

Maternal education level (years of education) was recorded in five categories: less than 7 years, 7–12 years, 13–14 years, 15–16 years, and more than 16 years of education. For the purposes of the analysis, this was then recategorized into three categories: less than 7 years to 12 years, between 13 and 16 years, and more than 16 years of education. The selection of this indicator was based on its identification as the best proxy indicator of socioeconomic status [19].

### 2.3. Anthropometric Measures

Body weight was measured in underwear and without shoes using an electronic scale (Type SECA 861 or SECA 813) to the nearest 0.1 kg, and body height was measured with a telescopic height instrument (Type SECA 225 or SECA 214) to the nearest 0.1 cm. Body mass index (BMI) was calculated as weight (kg) divided by squared height (m^2^). BMI z-scores (zBMI) were computed to classify children as being of a normal weight, being overweight, or being obese, for which the Cole et al. criteria were considered [20]. The intra- and interobserver reliability for weight and height was excellent (greater than 99% and 98%) in all participating countries [21].

### 2.4. Diet Assessment

Food and beverage consumption was assessed via a parentally reported semiquantitative food frequency questionnaire (FFQ) [22]. Low to moderate relative validity was observed, which varied by food and beverage group [23]. Estimated correlations ranged from 0.52 to 0.79. Food and beverage consumption was expressed as the number of portions per week. In the FFQ, 37 items were included, and in the current analysis they were merged into 21 groups according to their nutritional content (the main nutrient being proteins, carbohydrates, or fats). Of those 21, 10 were chosen and entered into the current analysis because they were considered to be associated with obesity development [24]: (1) water; (2) fizzy drinks (soft drinks and light drinks); (3) fresh fruit juices and packed juices; (4) dried, canned, and fresh fruits; (5) dairy products (milk, yogurt, and cheese); (6) sweets (chocolate and chocolate spreads, cakes, biscuits, and pastries); (7) desserts (smoothies, milk-based desserts, and sugar desserts); (8) meat and processed meat; (9) salty snacks; and (10) pasta and rice.

### 2.5. Physical Activity

In all of the countries except Belgium, PA was assessed by means of pedometers (Omron Walking Style Pro pedometers (HJ-720IT-E2)) assessing the number of steps per day. In Belgium, steps were measured using ActiGraph (Pensacola, FL, USA) accelerometers. Step counts from the accelerometers and pedometers were comparable. Evidence of their validity in preschool children indicated high correlations (daily, *r* = 0.89). In addition, evidence has suggested that the Omron Walking Style Pro pedometer is a valid and accurate measure to assess preschoolers’ steps per hour [25]. The devices were worn on the right hip (secured by an elastic waistband) for six consecutive days, including two weekend days [22]. The steps were further categorized into two categories, including ≥11,500 steps per day (if children followed the PA recommendations) and <11,500 steps per day (if children did not follow the PA recommendations). The selected step count cut-off was based on Reilly et al. [26] and De Craemer et al. [11].

### 2.6. Screen Time

Data on children’s screen time was collected via a standardized proxy-administered parental questionnaire (i.e., the Primary Caregivers’ Questionnaire). Screen time was used as a proxy indicator of sedentary behavior. The behaviors assessed included watching TV and DVDs and playing computer/video games. Parents/caregivers reported frequency for both weekdays and weekend days. The frequency categories included “never”, “less than 30 min/day”, “30 min to 1 h/day”, “1–2 h/day”, “3–4 h/day”, “5–6 h/day”, “7–8 h/day”, “8 h/day”, and “more than 8 h/day”. Average hours per day of TV/video viewing and personal computer use (separately for weekdays and weekend days) were summed up to obtain the screen time. To obtain the daily screen time, the average minutes per day, both for week- and weekend days, were summed up and divided by 7 days. The answers were further aggregated into two categories, including ≤1 h per day (if children followed the recommendations) and >1 h per day (if children did not follow the recommendations). These categories were based on the Australian and Canadian sedentary behavior recommendations, which state that preschool children should limit their screen time to a maximum of 1 h per day [9,27,28].

### 2.7. Statistical Analysis

**Figure 1:**
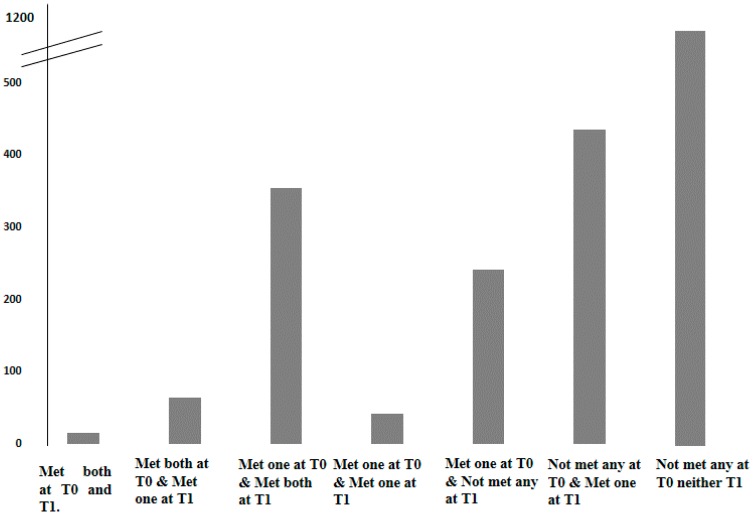
Proportion of European preschool children in each group. Groups are according to whether they met physical activity (PA) and/or screen time (ST) recommendations.

Statistical analyses were performed using Statistical Package for the Social Sciences (version 21.0; SPSS, Inc., Chicago, IL, USA). Analysis was done for the whole sample, as there were no differences by sex in all of the included variables as tested using a *t*-test for continuous variables and a chi-squared test for categorical variables. In order to evaluate possible changes in the adherence to both behaviors (ST and PA) between T0 and T1, seven groups were established, reflecting differential combinations of meeting or not meeting the ST and/or PA recommendations. Figure 1 shows the seven groups derived from possible combinations of ST and/or PA recommendations. Two of them included children who got worse in their behaviors from T0 to T1 (meeting both recommendations at T0 and meeting one of the recommendations at T1; meeting one of the recommendations at T0 and not meeting any recommendations at T1). Two groups included children who improved in their behaviors from T0 to T1 (not meeting any recommendations at T0 and meeting one of the recommendations at T1; meeting one of the recommendations at T0 and meeting both recommendations at T1). The last three groups included children who maintained their behaviors from T0 to T1 (meeting both recommendations at T0 and T1; meeting one of the recommendations at T0 and T1; and not meeting any recommendations at T0 and T1). After establishing the potential combinations of ST and PA recommendations, a lineal mixed effects model with random effects for country and food consumption at T1 as predictor variables and z-BMI, maternal education, and intervention versus control region at T1 as covariates were analyzed. Marginal means and standard deviations (SE) were used to show differences in food and beverage consumption by PA and ST recommendation combinations. All statistical tests and corresponding *p*-values lower than 0.05 were considered statistically significant.

## 3. Results

Table 1 presents descriptive information on age, gender, BMI categories, z-BMI scores, maternal education, and country for the total sample, both at the T0 and T1 periods. According to BMI, 2.8% of the sample at T0 was obese and 10.6% was overweight, while 3.2% of the sample at T1 was obese and 10.3% was overweight.

Table 2 presents the proportion of the sample adhering to PA and ST recommendations for preschool children at both time points. Only 12.4% of children at T0 and 8.8% at T1 met the PA recommendation. In terms of ST, 30.2% of children at T0 and 27.7% at T1 met the recommendations.

**Figure 2:**
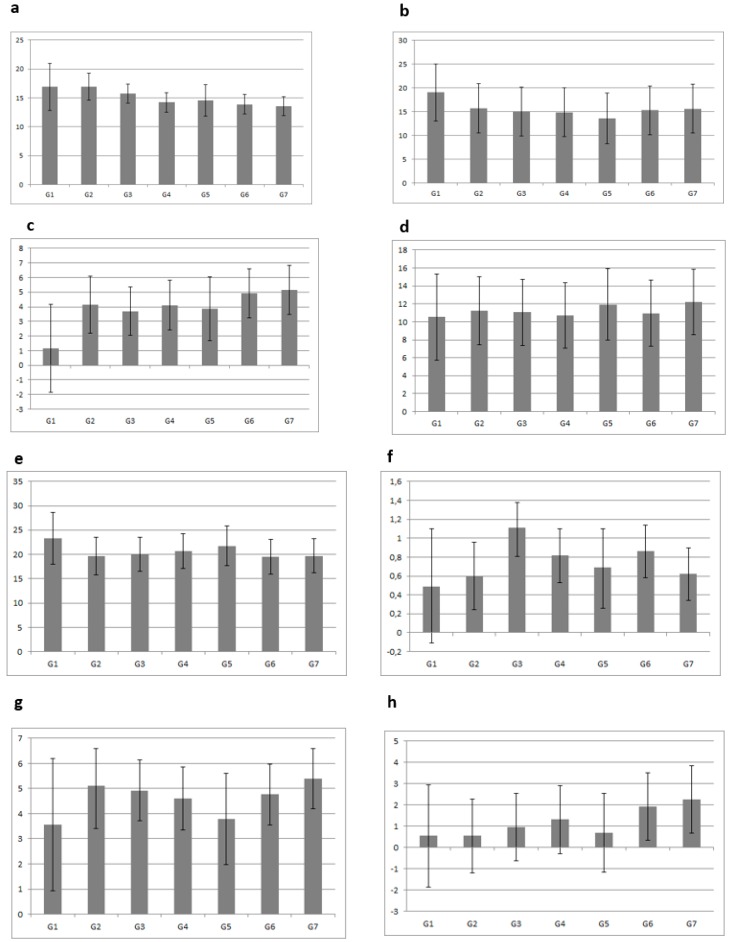
Marginal means and SD of food and beverage consumption (number of portions per week) according to PA and ST recommendations. G1: met both recommendations at T0 and met both recommendations at T1; G2: met both recommendations at T0 and met one of the recommendations at T1; G3: met one of the recommendations at T0 and met both recommendations at T1; G4: met one of the recommendations at T0 and met one of the recommendations at T1; G5: met one of the recommendations at T0 and did not meet any recommendations at T1; G6: did not meet any recommendations at T0 and met one of the recommendations at T1; G7: did not meet any recommendations at T0 and did not meet any recommendations at T1. Adjusted for maternal education, body mass index z-score at T1, sex, and center. Abbreviations: PA, physical activity; ST, screen time; SD, standard deviation; T0, baseline period; T1, follow-up period; F & V, fruits and vegetables; dairy products (milk, yogurt and cheese); desserts (smoothies, milk-based desserts and sugar desserts); sweets (chocolate and chocolate spreads, cakes, biscuits and pastries); juices (fresh fruit juices and packed juices); fizzy drinks (soft drinks and light drinks). (**a**) F & V, (**b**) dairy products, (**c**) dessert, (**d**) sweets, (**e**) water, (**f**) salty snacks, (**g**) juices, (**h**) fizzy drinks.

Derived from the general linear model, Figure 2 presents the marginal means and SDs of eight food and beverage groups (F & V, dairy products, desserts, sweets, water, salty snacks, juices, and fizzy drinks), according to several grouping combinations for meeting or not meeting PA and ST recommendations at baseline (T0) and follow-up (T1). In general, preschool children who met both recommendations (PA and ST) consumed less dessert (Figure 2c), sweets (Figure 2d), salty snacks (Figure 2f), juices (Figure 2g), and fizzy drinks (Figure 2h) and more F & V (Figure 2a), dairy products (Figure 2b), and water (Figure 2e). In contrast, preschool children who failed to meet the recommendations over time had a higher consumption of desserts (Figure 2c), sweets (Figure 2d), juices (Figure 2g), and fizzy drinks (Figure 2h), and lower consumption of F & V (Figure 2a) and water (Figure 2e).

Table 3 presents the associations between several grouping combinations of meeting or not meeting the PA and ST recommendations at T0 and at follow-up T1 and food and beverage consumption at follow up (T1). Seven possible combinations were identified. Approximately half of the sample (50.4%) did not meet either the PA or the ST recommendations in either period. With the opposite, only 0.6% of the sample met both PA and ST recommendations at both T0 and T1. Those who did not meet either recommendation at either time point (T0 and T1) were used as the reference group for analysis. Those children who met both recommendations at T0 and T1 consumed significantly fewer milk-based desserts and salty snacks in comparison to those who did not meet either recommendation at either time point. Those who met both recommendations at T0 and only one at T1 had a significantly lower consumption of fizzy drinks and salty snacks and higher consumption of F & V in comparison to the reference group. In addition, those children who met one of the recommendations at T0 and T1 had a significantly lower consumption of fizzy drinks, sweets, desserts, and salty snacks, and higher consumption of F & V. Those children who did not adhere to the recommendations at T0 and met one of them at T1 had a lower consumption of fizzy drinks, juices, sweets, desserts, and salty snacks in comparison to the reference group. At the same time, those children who met one of the recommendations at T0 and both at T1 had a lower consumption of fizzy drinks, juices, and salty snacks in comparison to not meeting either recommendation at either time point. Lastly, a significantly low consumption of juices, sweets, and salty snacks was observed in those children who met one of the recommendations at T0 and did not meet any recommendations at T1 in comparison to those who did not meet the PA and ST recommendations over time.

## 4. Discussion

In this study, associations between lifestyle behaviors, i.e., PA and ST and food and beverage consumption in preschool children, were investigated. The novelty of this report included examining adherence to both PA and ST recommendations across two time points and its relationship with food and beverage consumption in a large sample of European preschool children. The main finding of our study suggests that meeting both PA and ST recommendations at T0 and T1 was associated with a high consumption of foods considered healthy (F & V and water) and a lower consumption of energy-dense products (fizzy drinks, sweets, desserts, and salty snacks). In addition, we also observed a low proportion of children adhering to both recommendations during the follow-up.

The high proportion of children who failed to meet individual PA and ST recommendations at both periods agreed with results from other longitudinal studies, which observed similar trends. To our knowledge, there is no study reporting the proportion of preschool children meeting both PA and ST recommendations at the same time. Studies focusing on PA have reported that the percentage compliance with MVPA recommendations for European children is generally low [29]. The Health Behavior in School-Aged Children (HBSC) Study found that only 26% of its sample spent at least 1 h per day in MVPA [30]. Regarding ST, the HBSC study reported that 39% of the children complied with screen time recommendations [31]. In European children, approximately one-third of the children failed to meet current screen time recommendations [32]. An Australian study with a follow-up of three years reported that less than 20% of the sample met the ST recommendations and that screen time increased over the three-year follow-up. In addition, in an Australian study, participants were less likely to meet ST recommended guidelines as they got older [33].

In our sample, we found low levels of PA and high levels of ST in preschool children. The preschool age is an important period, as lifestyle behaviors such as PA and ST are established. However, different studies have observed that PA decreases during early childhood and adolescence [34,35] and even more so during the transition period from adolescence to adulthood [35,36]. Regarding ST, studies have observed an increase in ST during early childhood and also during the transition from primary to secondary school [37]. Both low PA and high ST have been associated with unfavorable body composition indicators such as BMI and waist circumference [38,39,40], both being determinants of obesity development in adulthood [33]. It has been suggested that ST, particularly TV, has an important role in the etiology of obesity due to its relationship with other unhealthy behaviors and its displacement of PA [41]. However, there is little evidence about the relationship between PA and ST on the one hand and food and beverage consumption on the other hand.

Previously, several cross-sectional studies analyzed the effects of PA or ST (alone) on food and beverage consumption. In European children (2–10 years old), boys and girls spending less time doing MVPA were more likely to consume fast food and less likely to consume vegetables and yogurt than those spending more time doing MVPA [13]. Similar associations were observed in European adolescents [42]: Those adolescents with the lowest PA levels consumed fewer fruits and dairy products compared to active adolescents. In a previous publication of the ToyBox study, those exceeding baseline ST recommendations had a higher consumption of energy-dense foods (sugar-based desserts, salty snacks, pastries, cakes, and biscuits) and beverages (fizzy drinks, sweet milk, and juices) than those complying with ST recommendations [12]. Another study reported that high TV viewing was related to less healthy food options (consumption of sweets and soft drinks) in children from different countries [43]. A study carried out in Brazil [44] in children less than two years old observed a positive association between time spent watching TV and the consumption of soft drinks. Santaliestra-Pasias et al. [45] reported that increased TV viewing and computer and internet use were associated with higher odds of sugar-sweetened beverage consumption and lower odds of fruit consumption in European adolescents. In an Australian adolescent sample, TV viewing was positively associated with energy-dense snack consumption and with higher availability of energy-dense snack foods at home [46].

There is consistent evidence to show that consumption of energy-dense foods is positively associated with low PA and high ST levels. However, research assessing the relationship between combinations of both PA and ST and food and beverage consumption is very scarce. Although high levels of both PA and ST have been observed in children [5], no studies have observed the combined effects of adherence to PA and ST recommendations on food and beverage consumption in preschool children.

This study had limitations. First, the generalizability of the findings is limited to the specific age group studied in the current study. The method used to assess PA was a pedometer, aside from Belgium. However, pedometers are not the gold standard in measuring preschool child PA, and therefore our results should be interpreted with caution. Nevertheless, the use of a pedometer provided objective information on PA, specifically in this age group. Information on food and beverage consumption and ST were collected via parental self-reported questionnaires, which are prone to over- or underreporting. However, the questionnaires used were developed/adapted and validated for the purposes of the study [22].

The main strengths of our study included the use of a large and culturally and socioeconomically diverse sample of preschool children from six different countries across Europe and its longitudinal design. Information through questionnaires was assessed via standardized and harmonized procedures [22].

## 5. Conclusions

This study examined the relationship between adherence to PA and ST recommendations and food and beverage consumption. In this sample of preschool children, we found that low PA levels and high ST were associated with an unhealthy food and beverage consumption profile. Preschool children and their parents should try to increase family time spent at activities promoting physical activity and to minimize the time they spend on screen time or being sedentary. In addition, public health interventions should focus on activities aimed at increasing movement in preschool children. Enabling healthier food and physical activity environments, together with the promotion of positive parental role modeling, should be prioritized to achieve higher rates of adherence to PA and ST recommendations.

## Figures and Tables

**Table 1:** Descriptive characteristics of European preschool children participating in the ToyBox-study.

		T0	T1	*P*-Figvalue
		Mean (SE)	Mean (SE)	
Age (years)		4.74 (0.4)	5.72 (0.4)	<0.001
Sex	Boys	1209 (52.1)	1210 (52.1)	0.152
	Girls	1111 (47.9)	1111 (47.9)	
BMI status *	Normal weight	1988 (85.7)	1980 (85.3)	
	Overweight	245 (10.6)	240 (10.3)	<0.001
	Obese	64 (2.8)	75 (3.2)	
zBMI		0.20 (1.0)	0.27 (1.0)	<0.001
		*n* (%)		
Maternal education	<7–12	337 (14.5)		
(years)	13–16	947 (40.8)		0.104
	>16	965 (41.6)		
Center	Belgium	522 (22.5)		
	Bulgaria	74 (3.2)		
	Germany	284 (12.2)		0.645
	Greece	317 (13.7)		
	Poland	688 (29.6)		
	Spain	435 (18.7)		

Abbreviations: BMI, body mass index; zBMI, body mass index z-score; T0, baseline period; T1, follow-up period; * BMI according to Cole’s cut-off [20].

**Table 2:** Number (%) of European preschool children participating in the ToyBox study that met or did not meet the physical activity and screen time recommendations at baseline (T0) and follow-up (T1) (*n* = 2321).

		T0 *n* (%)	T1 *n* (%)
Physical activity recommendations *	<11,500 steps/day	2033 (87.6)	2117 (91.2)
	≥11,500 steps/day	288 (12.4)	204 (8.8)
Screen time recommendations **	≤1 h/day	701 (30.2)	644 (27.7)
	>1 h/day	1620 (69.8)	1670 (72.3)

* Recommendations according to Reilly et al. [26] and De Craemer et al. [11] ** Recommendations on healthy eating and physical activity guidelines for early childhood settings in Australia [9,27,28].

**Table 3:** Results of the lineal mixed effects model between food consumption and adherence to physical activity and screen time recommendations at baseline (T0) and follow-up (T1) periods (*n* = 2321).

PA and ST Recommendations at T0	PA and ST Recommendations at T1	*n* %	Water	Fizzy drinks	Juices	F & V	Dairy Products	Sweets	Desserts	Meat and Processed Meat	Salty Snacks	Pasta and Rice
			β	β	β	β	β	β	β	β	β	β
			(95% CI)	(95% CI)	(95% CI)	(95% CI)	(95% CI)	(95% CI)	(95% CI)	(95% CI)	(95% CI)	(95% CI)
Met both recommendations	Met both recommendations	15 (0.6)	3.41	−1.65	−1.84	3.32	3.43	−1.69	−4.03	−2.38	−0.60	0.49
			(−1.22; 8.04)	(−3.71; 0.41)	(−4.32; 0.65)	(−0.62; 7.25)	(−0.77; 7.63)	(−5.43; 2.04)	(−6.75; −1.29) *	(−5.06; 0.29)	(−1.18; −0.02) *	(−0.51; 1.49)
Met both recommendations	Met one of the recommendations	64 (2.8)	0.25	−1.75	−0.38	3.42	0.078	−0.93	−1,04	−0.33	−0.51	−0.26
			(−2.16; 2.67)	(−2.80; −0.69) *	(−1.67; 0.90)	(1.41; 5.44) *	(−2.07; 2.23)	(−2.84; 0.98)	(−2.44; 0.35	(−1.66; 0.99)	(−0.80; −0.20) *	(−0.77; 0.25)
Met one of the recommendations	Met both recommendations	354 (15.3)	1.76	−1.47	−1.60	0.99	−2.07	−0.32	−1.32	−0.10	−0.41	−0.09
			(−1.09; 4.62)	(−2.78; −0.16) *	(−3.18; −0.02) *	(−1.50; 3.49)	(−4.73; 0.59)	(−2.69; 2.05)	(−3.05; 0.40)	(−1.77; 1.56)	(−0.78; −0.04) *	(−0.73; 0.55)
Met one of the recommendations	Met one of the recommendations	41 (1.8)	0.58	−1.34	−0.47	2.29	−0.58	−1.15	−1,45	−0.56	−0.49	0.01
			(−0.49; 1.66)	(−1.82; −0.86) *	(−1.05; 011)	(1.36; 3.20) *	(−1.56; 0.40)	(−2.03; −0.27) *	(−2.08; −0.81) *	(−1.17; 0.05)	(−0.62; −0.35) *	(−0.22; 0.25)
Met one of the recommendations	Did not meet any recommendations	241 (10.4)	0.07	−0.34	−0.63	0.38	−0.34	−1.24	−0.24	−0.28	−0.24	−0.21
			(−1.06; 1.21)	(−0.85; 0.16)	(−1.24; −0.01) *	(−0.58; 1.34)	(−1.37; 0.69)	(−2.15; −0.31) *	(−0.90; 0.43)	(−0.92; 0.35)	(−0.38; −0.09) *	(−0.46; 0.03)
Did not meet any recommendations	Met one of the recommendations	436 (18.8)	1.05	−0.97	−0.78	0.67	−0.74	−1.46	−1.05	−0.57	−0.29	0.03
			(−0.30; 2.41)	(−1.55; −0.38) *	(−1.49; −0.07) *	(−0.45; 1.79)	(−1.92; 0.45)	(−2.52; −0.40) *	(−1.82; −0.27) *	(−1.31; 0.17)	(−0.46; −0.12) *	(−0.26; 0.31)
Did not meet any recommendations	Did not meet any recommendations	1170 (50.4)	REF	REF	REF	REF	REF	REF	REF	REF	REF	REF

Adjusted for maternal education, body mass index z-score at T1, sex, and center. Abbreviations: PA, physical activity; ST, screen time; REF, reference group not meeting any recommendations at either T0 or T1; CI, confidence interval; fizzy drinks (soft drinks and light drinks); juices (fresh fruit juices and packed juices); F & V, fruits and vegetables; dairy products (milk, yogurt, and cheese); sweets (chocolate and chocolate spreads, cakes, biscuits, and pastries); desserts (smoothies, milk-based desserts, and sugar desserts). * *P* < 0.001.

## References

[1] (1999). Obesity: Preventing and Managing the Global Epidemic: Report of a WHO Consultation.

[2] Dietz W.H. (1998). Health consequences of obesity in youth: Childhood predictors of adult disease. PEDIATRICS.

[3] Van Stralen M.M., Velde S.J.T., Van Nassau F., Brug J., Grammatikaki E., Maes L., De Bourdeaudhuij I., Verbestel V., Galcheva S., Iotova V. (2012). Weight status of European preschool children and associations with family demographics and energy balance-related behaviours: A pooled analysis of six European studies. Obes. Rev..

[4] Paes V.M., Hesketh K., O’Malley C., Moore H., Summerbell C., Griffin S., Van Sluijs E.M.F., Ong K.K., Lakshman R. (2015). Determinants of sugar-sweetened beverage consumption in young children: A systematic review. Obes. Rev..

[5] Santaliestra-Pasías A.M., Mouratidou T., Reisch L., Pigeot I., Ahrens W., Mårild S., Molnár D., Siani A., Sieri S., Tornatiris M. (2015). Clustering of lifestyle behaviours and relation to body composition in European children. The IDEFICS study. Eur. J. Clin. Nutr..

[6] Kimm S.Y., Glynn N.W., Obarzanek E., Kriska A.M., Daniels S.R., Barton B.A., Liu K. (2005). Relation between the changes in physical activity and body-mass index during adolescence: A multicentre longitudinal study. Lancet.

[7] Miguel-Berges M.L., Reilly J.J., Moreno Aznar L.A., Jimenez-Pavon D. (2017). Associations Between Pedometer-Determined Physical Activity and Adiposity in Children and Adolescents: Systematic Review. Clin. J. Sport Med..

[8] Brug J., Van Stralen M.M., Velde S.J.T., Chinapaw M.J.M., De Bourdeaudhuij I., Lien N., Bere E., Maskini V., Singh A.S., Maes L. (2012). Differences in Weight Status and Energy-Balance Related Behaviors among Schoolchildren across Europe: The ENERGY-Project. PLoS ONE.

[9] Australian Department of Health and Aging Get Up and Grow: Healthy Eating and Physical Activity for Early Childhood.

[10] Tudor-Locke C., Craig C.L., Beets M.W., Belton S., Cardon G.M., Duncan S., Hatano Y., Lubans D.R., Olds T.S., Raustorp A. (2011). How many steps/day are enough? for children and adolescents. Int. J. Behav. Nutr. Phys. Act..

[11] De Craemer M., De Decker E., De Bourdeaudhuij I., Verloigne M., Manios Y., Cardon G. (2014). The translation of preschoolers’ physical activity guidelines into a daily step count target. J. Sports Sci..

[12] Miguel-Berges M.L., Santaliestra-Pasias A.M., Mouratidou T., Androutsos O., De Craemer M., Pinket A.-S., Birnbaum J., Koletzko B., Iotova V., on behalf of the ToyBox-study Group (2016). Associations between food and beverage consumption and different types of sedentary behaviours in European preschoolers: The ToyBox-study. Eur. J. Nutr..

[13] Santaliestra-Pasías A.M., Dios J.E.L., Sprengeler O., Hebestreit A., De Henauw S., Eiben G., Felső R., Lauria F., Tornaritis M., Veidebaum T. (2018). Food and beverage intakes according to physical activity levels in European children: The IDEFICS (Identification and prevention of Dietary and lifestyle induced health EFfects In Children and infantS) study. Public Health Nutr..

[14] Fernández-Alvira J.M., Mouratidou T., Bammann K., Hebestreit A., Barba G., Sieri S., Reisch L., Eiben G., Hadjigeorgiou C., Kovacs E. (2017). Parental education and frequency of food consumption in European children: The IDEFICS study. Public Health Nutr..

[15] Miguel-Berges M.L., Zachari K., Santaliestra-Pasias A.M., Mouratidou T., Androutsos O., Iotova V., Galcheva S., De Craemer M., Cardon G., Koletzko B. (2017). Clustering of energy balance-related behaviours and parental education in European preschool children: The ToyBox study. Br. J. Nutr..

[16] Androutsos O., Apostolidou E., Iotova V., Socha P., Birnbaum J., Moreno L., De Bourdeaudhuij I., Koletzko B., Manios Y. (2014). Process evaluation design and tools used in a kindergarten-based, family-involved intervention to prevent obesity in early childhood. The ToyBox-study. Obes. Rev..

[17] Manios Y. (2012). The ‘ToyBox-study’ obesity prevention programme in early childhood: An introduction. Obes. Rev..

[18] Manios Y., Androutsos O., Katsarou C., Iotova V., Socha P., Geyer C., Moreno L., Koletzko B., De Bourdeaudhuij I. (2014). Designing and implementing a kindergarten-based, family-involved intervention to prevent obesity in early childhood: The ToyBox-study. Obes. Rev..

[19] Nixon C.A., Moore H.J., Douthwaite W., Gibson E.L., Vögele C., Kreichauf S., Wildgruber A., Manios Y., Summerbell C. (2012). Identifying effective behavioural models and behaviour change strategies underpinning preschool- and school-based obesity prevention interventions aimed at 4–6-year-olds: A systematic review. Obes. Rev..

[20] Cole T.J., Bellizzi M.C., Flegal K.M., Dietz W.H. (2000). Establishing a standard definition for child overweight and obesity worldwide: International survey. BMJ.

[21] De Miguel-Etayo P., Mesana M.I., Cardon G., De Bourdeaudhuij I., Góźdź M., Socha P., Lateva M., Iotova V., Koletzko B.V., Duvinage K. (2014). Reliability of anthropometric measurements in European preschool children: The ToyBox-study. Obes. Rev..

[22] Mouratidou T., Miguel M.L., Androutsos O., Manios Y., De Bourdeaudhuij I., Cardon G., Kulaga Z., Socha P., Galcheva S., Iotova V. (2014). Tools, harmonization and standardization procedures of the impact and outcome evaluation indices obtained during a kindergarten-based, family-involved intervention to prevent obesity in early childhood: The ToyBox-study. Obes. Rev..

[23] Mouratidou T., Graffe M.I.M., Huybrechts I., De Decker E., De Craemer M., Androutsos O., Manios Y., Galcheva S., Lateva M., Gurzkowska B. (2019). Reproducibility and relative validity of a semiquantitative food frequency questionnaire in European preschoolers: The ToyBox study. Nutrition.

[24] Commission of the European Communities (2005). Green Paper: Promoting Healthy Diets and Physical Activity: A European Dimension for the Prevention of Overweight, Obesity, and Chronic Disease. https://publications.europa.eu/en/publication-detail/-/publication/fb6264c8-c756-47c4-944d-6d10bc9fce10/language-en.

[25] De Craemer M., De Decker E., Santos-Lozano A., Verloigne M., De Bourdeaudhuij I., Deforche B., Cardon G. (2015). Validity of the Omron pedometer and the actigraph step count function in preschoolers. J. Sci. Med. Sport.

[26] Reilly J.J., Coyle J., Kelly L., Burke G., Grant S., Paton J.Y. (2003). An Objective Method for Measurement of Sedentary Behavior in 3- to 4-Year Olds. Obes. Res..

[27] Leblanc A.G., Choquette L., Dillman C., Duggan M., Gordon M.J., Hicks A., Kho M.E., Latimer-Cheung A.E., Murumets K., Okely A.D. (2012). Canadian Sedentary Behaviour Guidelines for the Early Years (aged 0–4 years). Appl. Physiol. Nutr. Metab..

[28] (2019). Guidelines on Physical Activity, Sedentary Behaviour and Sleep for Children under 5 Years of Age.

[29] Konstabel K., Veidebaum T., Verbestel V., Moreno L.A., Bammann K., Tornaritis M., Eiben G., Molnár D., Siani A., on behalf of the IDEFICS consortium (2014). Objectively measured physical activity in European children: The IDEFICS study. Int. J. Obes..

[30] Inequalities in Young People’s Health: Health Behaviour in School-Aged Children (HBSC) Study. http://www.euro.who.int/__data/assets/pdf_file/0005/53852/E91416.pdf.

[31] Sigmundová D., Sigmund E., Bucksch J., Baďura P., Kalman M., Hamřík Z. (2017). Trends in Screen Time Behaviours in Czech Schoolchildren between 2002 and 2014: HBSC Study. Cent. Eur. J. Public Health.

[32] Santaliestra-Pasias A.M., Mouratidou T., Verbestel V., Bammann K., Molnar D., Sieri S., Siani A., Veidebaum T., Marild S., Lissner L. (2014). Physical activity and sedentary behaviour in European children: The IDEFICS study. Public Health Nutr..

[33] Hesketh K., Wake M., Graham M., Waters E. (2007). Stability of television viewing and electronic game/computer use in a prospective cohort study of Australian children: Relationship with body mass index. Int. J. Behav. Nutr. Phys. Act..

[34] Dumith S.C., Gigante D.P., Domingues M.R., Kohl H.W. (2011). Physical activity change during adolescence: A systematic review and a pooled analysis. Int. J. Epidemiol..

[35] Jones R.A., Hinkley T., Okely A.D., Salmon J. (2013). Tracking Physical Activity and Sedentary Behavior in Childhood: A Systematic Review. Am. J. Prev. Med..

[36] Telama R. (2009). Tracking of Physical Activity from Childhood to Adulthood: A Review. Obes. Facts.

[37] Pearson N., Haycraft E., Johnston J.P., Atkin A.J. (2017). Sedentary behaviour across the primary-secondary school transition: A systematic review. Prev. Med..

[38] Tremblay M.S., Leblanc A.G., Kho M.E., Saunders T.J., Larouche R., Colley R.C., Goldfield G., Gorber S.C. (2011). Systematic review of sedentary behaviour and health indicators in school-aged children and youth. Int. J. Behav. Nutr. Phys. Act..

[39] Biddle S.J., Petrolini I., Pearson N. (2014). Interventions designed to reduce sedentary behaviours in young people: A review of reviews. Br. J. Sports Med..

[40] Strong W.B., Malina R.M., Blimkie C.J., Daniels S.R., Dishman R.K., Gutin B., Hergenroeder A.C., Must A., Nixon P.A., Pivarnik J.M. (2005). Evidence Based Physical Activity for School-age Youth. J. Pediatr..

[41] Birch L.L., Davison K.K. (2001). Childhood overweight: A contextual model and recommendations for future research. Obes. Rev..

[42] Ottevaere C., Huybrechts I., Béghin L., Cuenca-Garcia M., De Bourdeaudhuij I., Gottrand F., Hagströmer M., Kafatos A., Le Donne C., Moreno L.A. (2011). Relationship between self-reported dietary intake and physical activity levels among adolescents: The HELENA study. Int. J. Behav. Nutr. Phys. Act..

[43] Vereecken C.A., Todd J., Roberts C., Mulvihill C., Maes L. (2006). Television viewing behaviour and associations with food habits in different countries. Publish Health Nutr..

[44] Jaime P.C., Prado R.R.D., Malta D.C. (2017). Family influence on the consumption of sugary drinks by children under two years old. Rev. Saude Publica.

[45] Santaliestra-Pasías A.M., Mouratidou T., Verbestel V., Huybrechts I., Gottrand F., Donne C.L., Cuenca-García M., Díaz L.E., Kafatos A., Manios Y. (2012). Food Consumption and Screen-Based Sedentary Behaviors in European Adolescents: The HELENA Study. Arch. Pediatr. Adolesc. Med..

[46] Pearson N., Biddle S.J., Williams L., Worsley A., Crawford D., Ball K. (2014). Adolescent television viewing and unhealthy snack food consumption: The mediating role of home availability of unhealthy snack foods. Public Health Nutr..

